# Ultrasensitive Detection of Bacteria by Targeting Abundant Transcripts

**DOI:** 10.1038/srep20393

**Published:** 2016-02-05

**Authors:** Xinhui Wang, Xinran Li, Shiwei Liu, Hang Ren, Mingjuan Yang, Yuehua Ke, Liuyu Huang, Chao Liu, Bo Liu, Zeliang Chen

**Affiliations:** 1 Institute of Zoonosis, Jilin University, 130062, Changchun, China; 2 Institute of Disease Control and Prevention, Academy of Military Medical Sciences, 100071, Beijing, China; 3 Wangjing Hospital, Academy of Traditional Chinese Medicine, 100102, Beijing, China; 4 School of Medicine, Shihezi University, 832003, Shihezi, China

## Abstract

Molecular detection assays are increasingly becoming routine diagnostic techniques for bacterial infection; however, their sensitivities are restricted by the low concentrations of bacteria in clinical samples. Here, we report a new paradigm for ultrasensitive detection of bacteria. The principle of this approach is that by choosing highly transcribed genes as signature sequences and detecting both DNA and its RNA transcripts, assay sensitivity can be greatly improved. First, signature genes with abundant transcripts were screened by RNA-Seq. We confirmed that RT-PCR efficiently amplifies both DNA and RNA, while PCR amplifies only DNA. Unexpectedly, we found that the RNA extraction efficiency is relatively low, while simplified denaturation was more appropriate for transcript detection. For highly transcribed genes, RT-PCR consistently generated lower cycle threshold (Ct) values than those of PCR. The sensitivity of RT-PCR targeting abundant transcripts could detect quantities as low as one bacterium, which was not possible using PCR. Amplification of different genes among several other common bacteria also confirmed that transcript detection by RT-PCR is more sensitive than is DNA detection by PCR. Therefore, abundant transcript detection represents a universal strategy for ultrasensitive detection of bacteria.

The ongoing outbreaks of MERS^1^ and Ebola^2^ and emerging pan-drug resistant bacterial pathogens^3^ remind us that infectious diseases are still a great public health problem. Rapid and sensitive detection of pathogens is of great importance for timely treatment and prevention of the spread of outbreaks^4^,^5^. Because of their specificity and sensitivity, molecular diagnostic methods are receiving increasing attention. Polymerase chain reaction (PCR) is the most popular technique for molecular detection of pathogens. For viral pathogens, their genomic nucleic acids exist in high abundance in clinical samples, making them easy to detect^6^. However, for bacterial pathogens, particularly those that survive in host cells, bacterial load is relatively low, making them challenging to detect^7^,^8^. Although highly sensitive methods have been developed for many bacterial pathogens, their sensitivities are still restricted by low concentrations of the pathogens in clinical samples^9^,^10^. Development of a new strategy that increases the detection sensitivity for bacterial pathogens will be of great value.

For an organism with DNA as its genetic material, genes are transcribed into RNA, which is translated into functional proteins. Expression levels of the encoded genes may vary depending on genes and environmental conditions. At present, all PCR assays detect bacterial genomic DNA, of which the copy number equals the number of bacteria. Since the number of bacteria in a clinical sample is predefined, detection sensitivity when targeting genomic DNA is also limited. In other words, if low numbers of bacteria are present in the sample, PCR sensitivity will be limited. Reverse-transcription PCR (RT-PCR) is an assay that detects RNA by reverse transcribing RNA into cDNA, which is then amplified by PCR. RT-PCR has been extensively used for RNA virus detection, and commercial RT-PCR kits are available only for RNA pathogens but not for DNA pathogens^11^. According to principle of RT-PCR, both the RNA and DNA in a sample can be amplified by RT-PCR. We hypothesized that if the RNA transcripts of a target gene are also detected, the assay sensitivity would be improved. Here, we report the development of a universal strategy for ultrasensitive detection of bacteria.

## Results and Discussions

**Figure 1:**
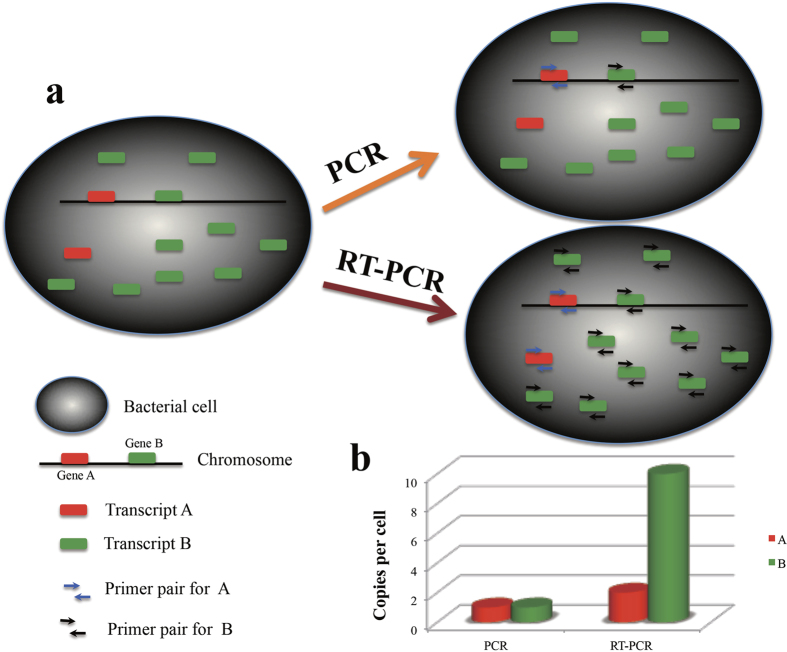
Principle of sensitive detection of bacteria by targeting abundant transcripts. (**a**) In a bacterial cell, one copy of gene A and nine copies of gene B are transcribed. PCR amplifies only genomic DNA, while RT-PCR could amplify both genomic DNA plus the transcripts. (**b**) The detected copies of gene A and B by PCR and RT-PCR. PCR would detect only one nucleic acid molecule, while RT-PCR would detect two and 10 nucleic acid molecules for A and B respectively.

Based on our hypothesis, we designed a new strategy to improve the sensitivity of bacterial detection. The principle is shown in Fig. 1. Gene A and gene B encoded by the genome (chromosome) are transcribed into one and nine RNA molecules, respectively. In terms of molecular detection, PCR can only detect genomic DNA, while RT-PCR can detect both genomic DNA and the transcribed RNA. For each bacterial cell, there is only one copy of each molecule that can be detected by PCR for genes A and B. However, when RT-PCR is used, there are two and 10 copies of each RNA molecule per cell to be detected for genes A and B, respectively. Since the sensitivity of PCR amplification is the same, reverse transcription of RNA into cDNA will increase the number of detectable DNA molecules and accordingly improve the sensitivity of the assay. Therefore, the selection of highly transcribed genes as signature sequences would increase the detection sensitivity. For the example of genes A and B, by selecting gene B as the signature gene, the sensitivity can be theoretically be improved fivefold using RT-PCR.

To test this hypothesis, *Brucella* was chosen as a test model. *Brucella* is a bacterial pathogen that survives in host cells and exists at low concentrations in clinical samples^12^. For development of a PCR assay, a signature sequence, which is unique to the genus or species, must first be identified. Based on the principle of the above hypothesis, an ideal signature gene should be species-specific, with a high level of transcription. Whole genome transcriptome sequencing represents a highly effective means to identify such genes. To screen for *Brucella*-specific genes with high transcription levels, all transcripts of *Brucella melitensis* 16 M were sequenced by RNA-Seq. The relative transcription levels defined as RPKM (Reads Per Kilobase of exon model per Million mapped reads) values were analyzed for all the annotated genes. Four genes with high transcription levels were chosen as signature genes for subsequent analysis (Table S1).

**Figure 2:**
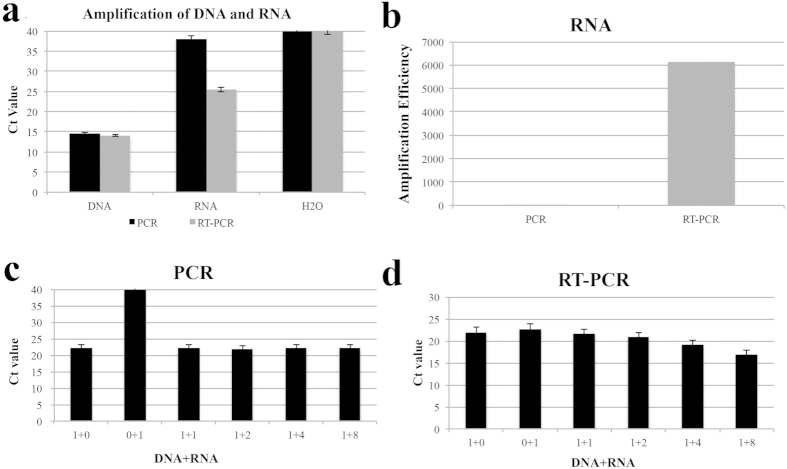
Differential amplification efficiencies of DNA and RNA by PCR and RT-PCR. DNA and RNA were extracted from *Brucella* culture and detected by PCR and RT-PCR (**a**); Efficiencies of RNA amplification by PCR and RT-PCR were compared (**b**); DNA was mixed with increasing quantities of RNA and detected by PCR (**c**) and RT-PCR (**d**).

The current consensus on PCR and RT-PCR is that PCR amplifies DNA and cDNA, while RT-PCR amplifies RNA. To test the amplification efficacies of PCR and RT-PCR for DNA and RNA, DNA and RNA extracted from *Brucella* cultures were detected with PCR and RT-PCR respectively. As shown in Fig. 2a, for the same DNA sample, PCR and RT-PCR generated similar Ct values, while for RNA samples, the RT-PCR Ct value was 10 lower than that of PCR. This indicated that RT-PCR could efficiently amplify both DNA and RNA, while PCR could efficiently amplify DNA but not RNA. The amplification efficiency of RNA by RT-PCR was about 6000 times that of PCR (Fig. 2b). To confirm further the difference in amplification efficiency of PCR and RT-PCR, DNA was mixed with increasing quantities of RNA and detected. With increasing quantities of RNA, the PCR Ct value did not change significantly (Fig. 2c), while the RT-PCR Ct values decreased (Fig. 2d). All these data confirmed that RT-PCR, but not PCR efficiently amplifies RNA.

**Figure 3:**
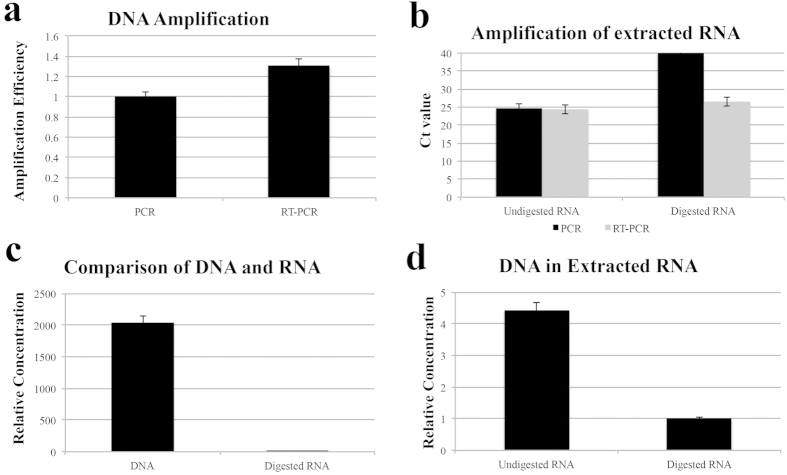
DNA contamination in extracted RNA. (**a**) Efficiency of DNA amplification by PCR and RT-PCR; (**b**) Amplification of undigested and digested RNA by PCR and RT-PCR; (**c**) Concentration comparison between DNA and digested RNA; (**d**) Relative concentration comparison between undigested and digested RNA.

Amplification efficiency of DNA by PCR and RT-PCR was calculated, and the results showed that efficiency of RT-PCR is about 1.31 times that of PCR (Fig. 3a). Extracted RNA samples are usually subjected to DNA enzyme digestion to remove contaminated DNA. Concentrations of DNA in crude and purified RNA were tested. When the crude RNA sample was amplified with PCR, the Ct value was 24.66, indicating significant DNA contamination in the RNA sample. After DNA digestion, no Ct value was observed for PCR, while for RT-PCR, the Ct value was increased from 24.35 to 26.4 (Fig. 3b). This indicated significant DNA contamination in the extracted RNA. Because DNA and RNA were isolated from equal quantities of bacterial sample, the Ct values represented the concentration of the templates. The concentration of DNA was about 2048 times that of the digested RNA (Fig. 3c). The concentration of undigested RNA (including both DNA and RNA) was four times higher than that of digested RNA (Fig. 3d). That is, RNA molecules comprised only 22.7% of the undigested RNA sample. We tested several RNA extraction techniques; the results showed that although they showed different extraction efficiencies, all the extractions resulted in significant loss of RNA (data not shown).

**Figure 4:**
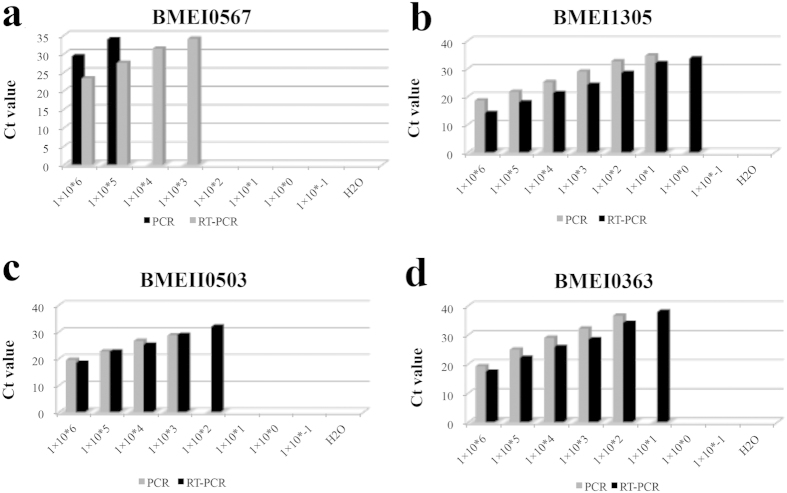
Sensitivity of selected target genes with PCR and RT-PCR. *Brucella* culture with defined concentration was heat denatured and serially diluted. Serially diluted cultures were subjected to detection by PCR and RT-PCR with primers for *BMEI0567* (**a**), *BMEI1305* (**b**), *BMEII0503* (**c**), and *BMEI0363* (**d**). Sensitivity of RT-PCR was consistently higher than that of PCR.

The above results showed that the RNA extraction efficiency is too low to be appropriate for RT-PCR detection. We speculated that this this might be due to the low efficiency of the assays or degradation of the sample during the extraction procedures. Therefore, we tested a simplified heat denaturation method for sample treatment. Bacterial cultures were suspended in DEPC-treated water and heat-denatured at 99 ^o^C. The supernatant was used as a template for PCR and RT-PCR. The RT-PCR Ct value was five times lower than the PCR Ct value (Fig. 4a). To further test whether RT-PCR was more sensitive than PCR, 10-fold serial dilutions of the lysates were detected. Results showed that for each dilution, the RT-PCR Ct values were consistently five lower than the PCR Ct values, and the lower detection limit of RT-PCR was lower than that of PCR (Fig. 4a). For signature gene *BMEI0567*, the RT-PCR assay can only detect 10^3^ CFU per reaction. To confirm the increased efficiency and to screen for a good candidate, another three genes, *BMEI1305, BMEII0503*, and *BMEI0363*, were selected and tested. As shown in Fig. 4(b–d), for any of these genes, the RT-PCR Ct value was one to five lower than that of PCR. The lower detection limit for RT-PCR was 10-fold lower than that of PCR for all of the three genes. However, the detection limits differed significantly. Sensitivity of RT-PCR for *BMEII0503* was limited to 100 CFU per reaction; for *BMEI0363*, 10 CFU; and for *BMEI1305*, 1 CFU. That is, RT-PCR of *BMEI1305* had the highest sensitivity, with a detection limit of 1 CFU, which was, in principle, the highest one for molecular detection.

Therefore, *BMEI1305* was an ideal signature candidate for RT-PCR detection of *Brucella*. To evaluate the sensitivity of RT-PCR for clinical sample detection, blood samples were collected from brucellosis patients and detected using PCR and RT-PCR assays. RT-PCR is identical to PCR with the exception of an initial step in which reverse transcriptase is used to transcribe an RNA sequence into its DNA complement. Blood samples were hemolyzed and mixed with DEPC-treated water (1:1) and heat-lysed for 5 min. Lysed samples were centrifuged and 2 μL of the supernatant was used for detection. PCR and RT-PCR were used to detect 10 sera-positive samples RT-PCR respectively. Four samples were found to be positive using PCR, while six were found to be positive using RT-PCR (Table 1). The PCR Ct values ranged from 35.4 to 38.5, while the RT-PCR Ct values ranged from 32.2 to 38.1. For each of the samples, the RT-PCR Ct value was about three cycles lower than that of PCR. For the two samples found positive only using RT-PCR, the Ct values were 37.2 to 38.1. This is reasonable, because if the Ct values were increased by three Ct, they would be outside the upper Ct value range for PCR. DNA sequencing of the amplification products confirmed that the two samples were true positives. These data indicated that RT-PCR could diagnose brucellosis with higher sensitivity than PCR and reduce the false negative rate (two in six, 33.3%).

To test whether this technique can be applied universally for bacterial detection, five other bacteria, including *Proteus mirabilis, Streptococcus pneumonia, Escherichia coli* UPEC, *Klebsiella pneumoniae,* and *Staphylococcus aureus*, were also detected. Candidate target genes with high transcription levels were selected based on previously reported expression profile studies. Bacterial cultures were heat lysed and detected using PCR and RT-PCR. As shown in Table 2, for the selected genes, the RT-PCR Ct values were lower than those of PCR. The decrease in Ct values varied with the different bacteria genus and target genes. Decreased Ct values ranged from less than one to higher than nine. That is, the most significant decrease would theoretically increase the sensitivity by about 1000 fold. These data confirmed that RT-PCR for sensitive detection of bacterial pathogens could be extended to other bacteria and be used as a universal strategy for bacterial detection.

## Concluding Remarks

In the present study, we have designed a new strategy for ultrasensitive detection of bacteria. This new approach uses the selection of highly transcribed signature genes and detection of their DNA and RNA. By using *Brucella* as a test model, we have demonstrated that some genes are transcribed at high levels, and RT-PCR could detect both DNA and RNA. We unexpectedly found that RNA extraction efficiency is low and is therefore not appropriate for sensitive RT-PCR detection. On the contrary, the simplified heat denaturation method is highly efficient in producing samples for sensitive detection by RT-PCR. The sensitivity of RT-PCR for both simulated and actual clinical samples is about 10-fold higher than that of PCR. The increased sensitivity of RT-PCR could improve the diagnostic performance and decrease the rate of false negative results in clinical samples, which is an important finding, since human brucellosis has been shown to be significantly misdiagnosed in some communities^13^. As experienced by the preliminary application, 33.3% (two in six) false negatives could be avoided. The principle of RT-PCR could also be extended to other molecular diagnostic methods, and the only modification required would be the addition of a reverse transcription step before amplification. Therefore, detection of bacteria by targeting abundant transcripts represents a universal approach to improve sensitivity over current methods. Our future work will include screening of highly transcribed signature genes and the development of new assays targeting these ultrasensitive biomarker molecules.

## Methods

*Brucella melitensis* 16 M was grown in tryptic soy broth (TSB) medium (OXOID) at 37 °C to an OD_600_~1.0, and then prepared for DNA and RNA extraction. Genomic DNA from 16 M was isolated by using TIANamp Bacteria DNA Kit (TIANGEN BIOTECH Co., Ltd.). RNA extraction was performed with TRIzol reagent (Invitrogen) according to the manufacturer’s recommendation. RNA was treated with Recombinant DNase I (Takara) to remove contamination from genomic DNA. RNA was subjected to RNA-Seq at BGI Shenzhen (Shenzhen, China). Relative transcription of genes by RNA-Seq was defined as Reads Per Kilobase of exon model per Million mapped reads (RPKM) value. Selected highly transcribed candidate genes were listed in Table S1. Candidate genes were BLASTed against GenBank to test sequence species-specificity. RNAs were reverse-transcribed into cDNA, using ImProm-II^TM^ Reverse Transcription System (Promega). For simplified sample treatment, bacteria culture was centrifuged at 12,000 g for 5 minutes, and then re-suspended in DEPC-treated water. The extracted DNA or cDNA was subjected to quantitative PCR (qPCR) using the SuperReal PreMix Plus (TIANGEN) with a 20-μl reaction system (which comprised of 10 μl 2 × SuperReal PreMix Plus, 1 μl of each primer (forward and reverse primers) and 3 μl of DEPC-tread H_2_O) on an IQ5 real-time PCR detection system (Bio-Rad). The reaction was performed using the following program: 5 min for the pre-denaturation step at 95 °C, followed by a 40 cycles of 20 s at 95 °C, 30 s at 55 °C, and 30 s at 72 °C. Primers used in the present study are listed in Table S2.

## Additional Information

**How to cite this article**: Wang, X. *et al.* Ultrasensitive Detection of Bacteria by Targeting Abundant Transcripts. *Sci. Rep.* **6**, 20393; doi: 10.1038/srep20393 (2016).

## Supplementary Material

Supplementary Information

## Figures and Tables

**Table 1:** Detection results of blood samples from brucellosis patients by PCR and RT-PCR.

**Sample**	**PAT**^a^	**SAT**^b^	**RT-PCR**^c^	**PCR**	**Sequencing confirmation**^d^
Bru01	0.01	1/200	35.2	38.5	Yes
Bru02	0.01	1/200	37.2	N/A	Yes
Bru03	0.01	1/200	34.6	37.2	Yes
Bru04	0.01	1/200	N/A	N/A	ND
Bru05	0.01	1/200	32.2	35.4	Yes
Bru06	0.04	1/100	N/A	N/A	ND
Bru07	0.01	1/100	33.6	36.8	Yes
Bru08	0.01	1/100	N/A	N/A	ND
Bru09	0.01	1/100	38.1	N/A	Yes
Bru10	0.01	1/100	N/A	N/A	ND

^a^PAT: plate agglutionation test, values lower than 0.04 are positives.

^b^SAT: standard tube agglutination test, values higher than 1/100 are positives.

^c^N/A: No value.

^d^ND: not determined.

**Table 2:** Sensitivity improvement by targeting abundant transcripts among different bacteria genus.

**Bacteria**	**Target Gene**	**PCR**	**RT-PCR**	**Ct value**
*Proteus mirabilis*	tufb	12.59	11.34	1.25
	trmd	12.62	12.84	−0.22
	atp	11.77	11.2	0.57
*Streptococcus pneumoniae*	ATPFB	32.35	24.83	7.52
	SRPL	30.77	21.05	9.72
	GSP24	29.62	16.21	13.41
*Klebsiella pneumoniae*	ompA	15.18	14.03	1.15
	cyoB	14.2	13.17	1.03
	cyoD	13.75	12.37	1.38
*Staphylococcus aureus*	HLSN	12	11.75	0.25
	KG	11.79	9.63	2.16
	PA	11.86	10.61	1.25
*E. coli UPEC*	rpsc	26.4	21.01	5.39
	rplD	25.64	16.34	9.3
	GAPA	24.88	21.13	3.75
