# Supplementary material for: Ultrasensitive Detection of Bacteria by Targeting Abundant Transcripts
Source: Sci Rep. 2016 Feb 5;6:20393. doi: 10.1038/srep20393 (PMC4742959; doi:10.1038/srep20393)
Supplement: Supplementary Information [file srep20393-s1.doc]

**Ultrasensitive Detection of Bacteria by Targeting Abundant Transcripts**

Xinhui Wang^1,2#^, Xinran Li^1,2#^, Shiwei Liu^3,#^, Hang Ren^2,#^, Mingjuan Yang^2^, Yuehua Ke^2^, Liuyu Huang^2^, Chao Liu^2^, Bo Liu^1,*^, Zeliang Chen^1,4*^

^1^ Institute of Zoonosis, Jilin University, 130062, Changchun, China

^2^ Institute of Disease Control and Prevention, Academy of Military Medical Sciences, 100071, Beijing, China

^3^ Wangjing Hospital, Academy of Traditional Chinese Medicine, 100102, Beijing, China

^4^ School of Medicine, Shihezi University, 832003, Shihezi, China

^#^ The authors contributed equally to this work.

^*^ Corresponding authors

Zeliang Chen, [zeliangchen@yahoo.com](mailto:zeliangchen@yahoo.com), Bo Liu, [Liuboy@hotmail.com](mailto:Liuboy@hotmail.com)

Supplementary Tables

Table S1 Transcription level of selected signature candidate genes

Table S2 Primers used in the present study

Table S1 Transcription level of selected signature candidate genes

| Gene ID | Length | RPKM average | Product |
| --- | --- | --- | --- |
| BMEII0503 | 417 | 4125.0 | hypothetical protein |
| BMEI0567 | 513 | 3126.5 | hypothetical protein |
| BMEI0363 | 348 | 2684.2 | tonb protein |
| BMEI1305 | 375 | 3286.8 | Porin |

Table S2 Primers used in the present study

| Primer name | Sequence |
| --- | --- |
| BMEI0567-RT-F | GGTTTGCTTTCTGTGGCACT |
| BMEI0567-RT-R | TACAGGGTTGCCTGATGTGA |
| BMEII0503-RT-F | ATGGTTCGTAAACAGCGGAC |
| BMEII0503-RT-R | AACGATTTCCTTGAGGGCTT |
| BMEI1305-RT-F | TACCTTCACCGGTTACCTCG |
| BMEI1305-RT-R | CGATGTGGTAGTTGGTCGTG |
| BMEI0027-RT-F | TATATCATCGTTGGAGGCGG |
| BMEI0027-RT-R | GATGACTTTCGCCTGCGTAT |
| BMEI0363-RT-F | GCCTTTGTGATCGACCCTT |
| BMEI0363-RT-R | TTACTTGAACAGAAGCGGCA |
| tufB-RT-F | AGGTCGTCAGGTTGGTGTTC |
| atpF-RT-F | GCAATCATTGAGTCTGCGAA |
| trmD-RT-F | GGAACTCCCTGCCATGATAA |
| tufB-RT-R | TTTTGCTTCCCACTCTGCTT |
| atpF-RT-R | AGCCAGCATAGCGACTTGTT |
| trmD-RT-R | ACAGTAACACTGCCGGAACC |
| cyoB-RT-F | GATGCCTTCTGGGAAATGAA |
| cyoD-RT-F | ATCATCCTGACGGTCATTCC |
| ompA-RT-F | TAACGATCAGCTTGGTGCTG |
| cyoB-RT-R | AATCCACGTCCTCGTCAAAG |
| cyoD-RT-R | CAACCTTCATCGGACTTGCT |
| ompA-RT-R | GCGTAGTTGCCTTTGGAGTC |
| rpsC-RT-F | GTACACGACGGAATGAAGCA |
| rplD-RT-F | CTTCGAAGTTGAATGCGTCA |
| gapA1-RT-F | CAAGACGCACCTCACAGAAA |
| rpsC-RT-R | GAGATTGAACGTGCTGCAAA |
| rplD-RT-R | AGGTACAATCCGTGCTCCAC |
| gapA1-RT-R | ACGTTGTGCACCACCATCTA |
| PA-RT-F | TATCATCCATGCGGACAAAA |
| KG-RT-F | GGCCAGCAAAGAAGAGACAC |
| hlsN-RT-F | GATTAGCCTTGCGTCTCTGG |
| PA-RT-R | CTGGCATTAAGTGGCGGTAT |
| KG-RT-R | ATGGACACCAGGCAAGAATC |
| hlsN-RT-R | AATCACCACCAGCATCAACA |
